# Novel and multifaceted regulations of photoperiodic flowering by phytochrome A in soybean

**DOI:** 10.1073/pnas.2208708119

**Published:** 2022-10-03

**Authors:** Xiaoya Lin, Lidong Dong, Yang Tang, Haiyang Li, Qun Cheng, Hong Li, Ting Zhang, Lixin Ma, Hongli Xiang, Linnan Chen, Haiyang Nan, Chao Fang, Sijia Lu, Jigang Li, Baohui Liu, Fanjiang Kong

**Affiliations:** ^a^Guangdong Key Laboratory of Plant Adaptation and Molecular Design, Guangzhou Key Laboratory of Crop Gene Editing, Innovative Center of Molecular Genetics and Evolution, School of Life Sciences, Guangzhou University, Guangzhou 510006, China;; ^b^The Innovative Academy of Seed Design, Key Laboratory of Soybean Molecular Design Breeding, Northeast Institute of Geography and Agroecology, Chinese Academy of Sciences, Harbin 150081, China;; ^c^National Key Laboratory of Crop Genetics and Germplasm Enhancement, National Center for Soybean Improvement, Jiangsu Collaborative Innovation Center for Modern Crop Production, Nanjing Agricultural University, Nanjing 210095, China;; ^d^State Key Laboratory of Plant Physiology and Biochemistry, College of Biological Sciences, China Agricultural University, 100193 Beijing, China

**Keywords:** phytochrome A, photoperiodic flowering, adaptation, soybean

## Abstract

Photoperiod is an important environmental cue. Plants can distinguish the seasons and flower at the right time through sensing the photoperiod. Soybean is a sensitive short-day crop, and the timing of flowering varies greatly at different latitudes, thus affecting yields. Soybean cultivars in high latitudes adapt to the long day by the impairment of two phytochrome genes, *PHYA3* and *PHYA2*, and the legume-specific flowering suppressor, *E1*. However, the regulating mechanism underlying phyA and E1 in soybean remains largely unknown. Here, we classified the regulation of the E1 family by phyA2 and phyA3 at the transcriptional and posttranscriptional levels, revealing that phyA2 and phyA3 regulate E1 by directly binding to LUX proteins, the critical component of the evening complex, to regulate the stability of LUX proteins. In addition, phyA2 and phyA3 can also directly associate with E1 and its homologs to stabilize the E1 proteins. Therefore, phyA homologs control the core flowering suppressor E1 at both the transcriptional and posttranscriptional levels, to double ensure the E1 activity. Thus, our results disclose a photoperiod flowering mechanism in plants by which the phytochrome A regulates LUX and E1 activity.

## Significance

Plants know the exact time of flowering by sensing the photoperiod. Flowering time is an important agronomic trait in crops. In order to ensure that crops maintain high yields in different latitudes, cultivars need to accurately adjust the flowering time of plants according to local photoperiod and environmental conditions. In many plants, phytochromes have been found to be involved in photoperiodic flowering, but the molecular mechanisms of how they control photoperiod flowering are not fully understood. Through a series of biochemical, molecular, and genetic analyses of soybean phytochrome A, we reveal a photoperiod flowering mechanism in plants by which the phytochrome A regulates LUX and E1 activity.

Plants perceive the day length or photoperiod as seasonal changes to integrate their intricate components and orchestrate the developmental and physiological processes to cope with the constantly changing environmental conditions. Flowering is the key milestone in plant development in which reproductive growth is initiated and it is therefore one of the most important determinants of crop adaptation and yield. One hundred years ago, Wightman Garner and Harry Allard made the first pioneer report on plant photoperiodism in a seminal paper, which prominently featured soybean and tobacco as model plants (1). Over the following decades, the physiological and molecular basis of photoperiodic flowering has been investigated and characterized in many plant species, with most detailed in *Arabidopsis* and rice, two representative long-day (LD) and short-day plants (SDP). An array of photoreceptors and intricate signaling pathways allow plants to convey the surrounding light and photoperiod information and synchronize an endogenous timekeeping system known as the circadian clock to determine flowering time.

The day length-specific expression of FLOWERING LOCUS T (FT) protein is essential for the proper timing of flowering in plants. In *Arabidopsis*, *FT* transcription is directly activated by CONSTANS (CO) transcriptional factor, in which the restriction of its protein activity to the long-day afternoon for proper *FT* induction, both circadian clock regulation of CO transcription and photoreceptor regulation of CO protein abundance are necessary (2). In particular, photoreceptors play the essential roles in these regulations of CO transcriptionally and posttranscriptionally to control photoperiod flowering (2). For instance, the red (R) light and thermosensor photoreceptor phytochrome B (phyB) and far-red (FR) light photoreceptor phyA antagonistically to control flowering by regulating the stability of CO protein in *Arabidopsis* (3–7). In the morning under the LD, phyB absorbs R light and interact with the RING finger-containing E3 ubiquitin ligase HOS1 (HIGH EXPRESSION OF OSMOTICALLY RESPONSIVE GENE 1) to promote degradation of the CO protein (8). However, in late afternoon under LD, phyA and blue light photoreceptors cryptochrome 1 (cry1), cry2 and FLAVIN-BINDING KELCH REPEAT F-BOX 1 (FKF1), all function to stabilize the CO protein. Among these photoreceptors, phyB and cry2 play the major roles of photoperiod flowering in *Arabidopsis* (2, 9, 10). In rice, phyB has larger effects on flowering than phyA and phyC which is proved by night break experiments (11). phyB promotes the protein degradation of EARLY FLOWERING 3 (ELF3), a core component of evening complex (EC) in circadian clock (12, 13), thereby releasing the suppressions of EC on two CCT-domain flowering repressors Grain number, Plant Height, and Heading date1 (Ghd7) and PSEUDO-RESPONSE REGULATOR37 (PRR37) to delay photoperiod flowering (14).

As the model plant for identification of photoperiodism in 1920, soybean is an extremely photoperiod sensitive SDP, and this sensitivity limits its latitudinal adaptation. Cultivated soybean domesticated from its progenitor (*Glycine soja*) in Huanghuaihai region in middle of China around 5000–8000 y ago (15). Since then, it has been expanded from its central origins to wide range of latitude worldwide. In high latitudes where day length becomes longer in summer, soybean must reduce the photoperiod sensitivity in order to flower and mature earlier before the first frost comes (16, 17). On the contrary, as soybeans expand to lower latitudes where it flowers very earlier and results in lower yields, soybean should extend its vegetative phase delaying flowering and maturity to maximize the yield (18). Therefore, to fine-tune soybean maturity and adaptation, a series of genetic loci or genes quantitatively participate in this regulation (19). Some of them have been extensively identified and molecular characterized (20–34). Of these identified genes in particular, *E1* is a legume-specific transcriptional factor that plays a central and integrated role in photoperiod flowering pathway (20). In addition, E3 and E4 have been identified as homologs of phytochrome A (*PHYA3* and *PHYA2*) genes, which were demonstrated as the major photoperiod receptors to control photoperiod flowering in soybean (22, 23, 35). Recently, it was reported that the complete impairment of circadian EC caused by double mutants of *lux1 lux2* leads to soybean photoperiod insensitivity between SD and LD (30). All these results suggest that the homologs of *PHYA*, *EC*, and *E1* are the major genetic players in the control soybean photoperiod sensitivity, flowering, and latitudinal adaptation, but the underlying regulatory networks among them remain largely unknown.

Here, we show that phyA other than phyB plays the critical role in soybean photoperiod flowering. phyA3 protein is relatively stable under all light conditions while phyA2 protein is extremely unstable, especially under R light. We then demonstrate that phyA3 and phyA2 proteins physically interact with LUX proteins, the core components of EC, and this interaction in turn promotes the degradation of LUXs, resulting in the up-regulation of *E1* expression and late flowering. We further confirm that *PHYA3* and *PHYA2* are largely genetically dependent on *LUX* genes. Most interestingly, we also found that phyA3 and phyA2 physically interacted with E1 and its homologs proteins to stabilize the E1 proteins. The phyA3/phyA2-E1 protein complex can directly bind to the E1-binding site of *FT2a* and *FT5a* promoter region to suppress their transcription. We also made 16 genetic materials of different combinations of *phyA3*, *phyA2*, *e1*, and *e1l* genes to illustrate that *PHYA3* and *PHYA2* are genetically dependent on *E1* family genes. In conclusion, our results suggest a photoperiod flowering pathway in soybean underlying phyA regulation through transcriptionally and posttranscriptionally manipulating a flowering suppressor of E1.

## Results

### Impairment of *PHYA2* and *PHYA3* Resulted in Altered Transcription of Photoperiodic Flowering Genes.

**Fig. 1.**
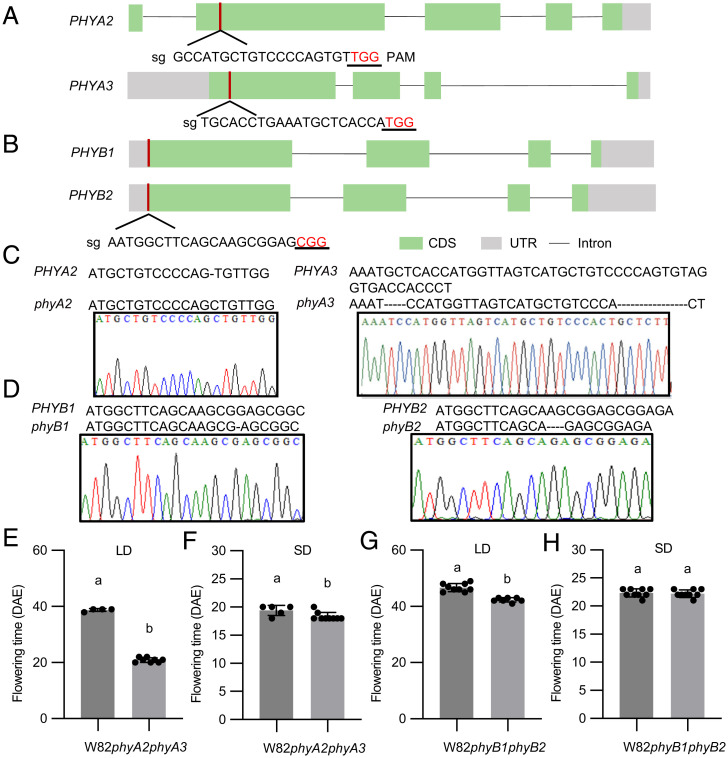
phyA was the main regulator of photoperiodic flowering in soybean. (*A*, *B*) The gene structure of soybean *PHYA2*, *PHYA3* molecules (*A*) and *PHYB1*, *PHYB2* (*B*). (*C*, *D*) The red bar indicates where the single guide RNA (sgRNA) is. CRISPR/Cas9-induced 5 + 16-bp deletion at target site for *phyA3*, 1-bp insertion at target site detected for *phyA2* (*C*) and 1-bp deletion at target site for *phyB1*, 4-bp deletion at target site for *phyB2* (*D*) by Sanger sequencing. Flowering time of wild-type plants (WT, W82) and homozygous mutant *phyA2 phyA3* (*E*, *F*), WT, *phyB1 phyB2* (*G*, *H*) mutants under LD conditions (16-h light/8-h dark) and SD (12-h light/12-h dark).

Soybean has a paleopolyploid genome and its genome undergone two rounds of duplications occurred at ∼59 and 13 million years ago, and ∼75% of soybean genes are present in multiple copies leading to high gene redundancy and diversification (36). For this reason, soybean possesses four homologs of *PHYA*, including *PHYA3* (E3) and *PHYA2* (E4), two homologs that were functionally characterized (16, 19). Soybean possesses two homologs of *PHYB*, which have not been studied before in soybean photoperiodic flowering. To better study the functions of *PHYA* and *PHYB*, we used CRISPR-Cas9 system and soybean genetic transformation to knockout *PHYA2*, *PHYA3*, and *PHYB1*, *PHYB2* in cultivar Williams 82 (W82) (Fig. 1 *A*–*D* and *SI Appendix*, Fig. S1 *A–C*). Knocking out of *PHYA2* and *PHYA3* leads to extremely early flowering comparing with wild-type W82 under LD, but minor differences under SD (Fig. 1 *E* and *F*), which was in accordance with previous results (28, 37). In contrast, knocking out of *PHYB1* and *PHYB2* resulted in only a few days of early flowering in soybean under LD and no difference in flowering under SD (Fig. 1 *G* and *H*), indicating that phyA, rather than phyB, has a major role in photoperiodic flowering in soybean. Next, quantitative RT-PCR analysis showed that the transcriptional levels of *E1* and its two homologs *E1la* and *E1lb* were dramatically reduced to nearly undetectable, whereas the expression of soybean two major homologs of *FLOWERING LOCUS T* (*FT*) *FT2a* and *FT5a* (37) were largely enhanced in *phyA2 phyA3* double mutants under LD conditions (*SI Appendix*, Fig. S2). In addition, some key circadian clock genes which were proved to control soybean flowering including *LUX*, *J*, *LHY*, *TOF11*, and *TOF12*, were also down-regulated in *phyA2 phyA3* plants (*SI Appendix*, Fig. S2). Taken together, these results indicate that phyA2 and phyA3 have a great regulation on *E1* family and florigen genes and also affect other circadian clock gene expression to regulate photoperiod flowering.

### The Homologs of Phytochrome A Are Differently Regulated by Light.

Previous results showed phyA3 and phyA2 together control the photoperiod sensitivity in low R:FR (38), while phyA3 controls photoperiod sensitivity in high R:FR (39), which suggested that they might undergo subfunctionalization to confer photoperiod sensitivity and flowering under different ratio of R:FR in soybean. In order to gain more insights of the protein accumulations of phyA3 and phyA2 under different light in soybean, we developed antibodies against phyA3 and phyA2, respectively. Like phytochromes in *Arabidopsis*, phyA3 and phyA2 proteins were detected under the dark conditions (*SI Appendix*, Fig. S3*A*). No bands were detected in *phyA3* natural mutant NIL-*PHYA2 phyA3* using phyA3 antibody (*SI Appendix*, Fig. S3*A*), while a lighter band was still detected in *phyA2* natural mutant NIL-*phyA2 PHYA3* using phyA2 antibody (*SI Appendix*, Fig. S3*A*), which indicated that phyA3 antibodies can specifically recognize phyA3 protein, but phyA2 antibody recognizes both phyA2 and its paralogue, phyA1.

We then examined whether phyA3 and phyA2 protein accumulation are regulated by different light. The W82 seedlings were grown in dark (D), FR light, R light, and blue (B) light for 3 d and harvested and then subjected to immunoblot. phyA3 and phyA2 proteins both accumulated in dark but accumulated differentially under different light conditions (*SI Appendix*, Fig. S3*B*). Notably, the accumulations of phyA3 proteins under FR and B light are similar to that under dark, which are much more abundant than that in R (*SI Appendix*, Fig. S3*B*). However, the accumulations of phyA2 protein are greatly affected by different light conditions, compared with that under D. phyA2 protein was not detected under R light, but weakly accumulated under FR and B light (*SI Appendix*, Fig. S3*B*). To further confirm that phyA2 and phyA3 proteins were indeed specifically regulated by different light, the W82 seedlings were first grown under D for 4 d, then transferred to different light. We observed that phyA3 proteins were relatively stable within 2 h of light exposure after transferring from dark to light regardless of different light conditions (*SI Appendix*, Fig. S3 *C–F*). However, the stability of phyA2 proteins were differently regulated under different light (*SI Appendix*, Fig. S3 *C–F*). phyA2 proteins down-regulated as soon as 5 min R light exposure and vanished within 30 min thereafter (*SI Appendix*, Fig. S3*E*). In addition, phyA2 proteins also down-regulated under B light after 30 min transferring from D (*SI Appendix*, Fig. S3*D*). Taken together, our data demonstrated that phyA3 proteins were more stabilized than phyA2 proteins under all light conditions, further indicated that phyA3 possessed major functions than phyA2 in the control of photoperiod flowering and sensitivity (16, 19). These results also implied that phyA2 and phyA3 underwent subfunctionalization after soybean genome duplication during its evolution.

### phyA2 and phyA3 Interacted with LUX to Mediate Its Degradation.

**Fig. 2.**
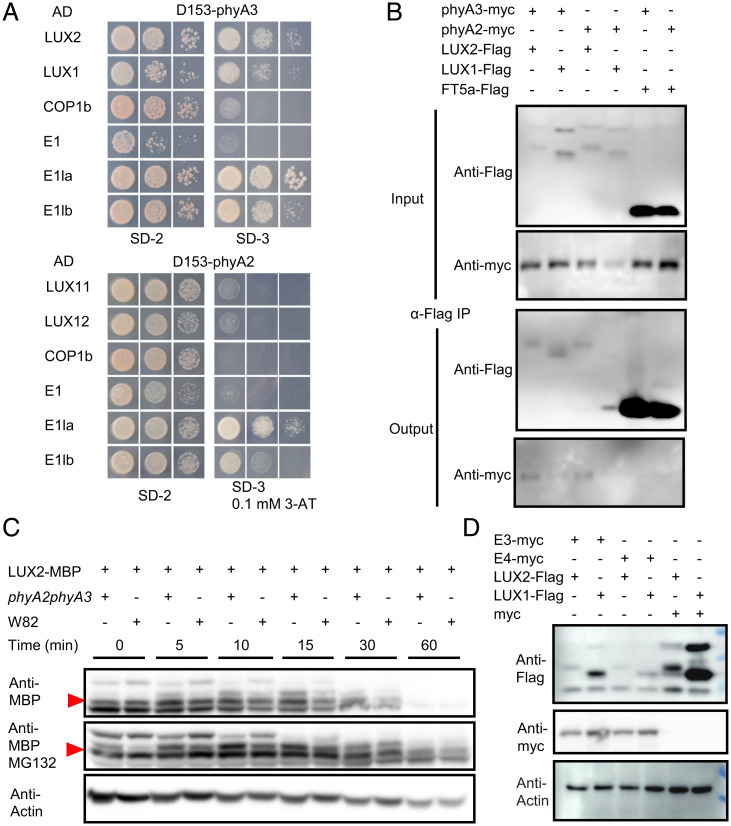
phyA2 and phyA3 interacted with and mediated the degradation of LUX1 and LUX2. (*A*) Yeast two-hybrid assays showing that phyA2 and phyA3 interacted with LUX1, LUX2, E1la, and E1lb. (*B*) Co-IP assays showing that phyA2 and phyA3 interacted with LUX1 and LUX2 in vivo. (*C*) Cell-free in vitro degradation system indicating that LUX2-MBP is stabilized in protein extracts from *phyA2 phyA3* plants. Anti-actin was used as a sample loading control. Since the other bands were inconsistent when initially added, the bands shown by the red triangles were used to compare strengths. (*D*) Transient expression in *Nicotiana benthamiana* leaves demonstrating that phyA2 and phyA3 mediate the degradation of LUX1 and LUX2 in plants. Anti-actin was used as a sample loading control.

To elucidate how phyA3 and phyA2 regulate transcription of *E1* family to control flowering, we then screened the interacting proteins with phyA3 and phyA2 using the full length of phyA3 and phyA2 (40). Interestingly, we identified two groups of proteins that interacted with phyA3 and phyA2 including LUXs and E1s (Fig. 2*A*), both of which were transcriptional factors and played essential roles in photoperiod flowering and sensitivity in soybean (30). Next, to determine which form (Pr or Pfr) of phyA3 and phyA2 associated with LUXs more strongly, phycocyanobilin was added to GAL4 yeast two hybrid system to allow the phytochromes to form Pr or Pfr forms after R light or D treatments. COP1, which was reported have no interaction with phyA in GAL4 Y2H system (41), was used as a negative control (Fig. 2*A*). We observed that both forms of phyA3 and phyA2 can interacted with LUXs in yeast (*SI Appendix*, Fig. S4), indicating that the interactions are not dependent on light. Previous reports also showed that phyB and phyA interacts with ELF3 in *Arabidopsis*, which is also a member of the evening complex, together with LUX (42, 43). Their interactions promoted us to test the protein interactions between phyA3 and phyA2 and J protein, the counterpart of ELF3 in soybean (28). Unlike the results from *Arabidopsis* (42), the protein interactions between phyA3, phyA2 and J failed in Y2H (*SI Appendix*, Fig. S5), implying different association mechanisms among those proteins in different plant species in which the interactions between PHYA and LUX proteins have not been identified previously in any plant species.

The protein interactions between phyA3, phyA2, and LUXs were further confirmed by the coimmunoprecipitation (co-IP) assay in tobacco leaves (Fig. 2*B*). We therefore asked if phyA3 and phyA2 could mediate LUXs degradation from these interactions in plants. Next, we used cell-free system to test the stability of LUX proteins incubated with the protein extracts from either the wild-type W82 or the double mutants of *phyA2 phyA3*. As we expected, 10 min after LUX proteins were added to the protein extracts, degradation of LUX was stronger in buffer containing W82 plant extracts than the mutants of *phyA2 phyA3* (Fig. 2*C* and *SI Appendix*, Fig. S6*A*). The presence of MG132 can slow down the rate of degradation and reduce the difference in the rate of degradation of LUX2 by W82 and *phyA2 phyA3* extracts (Fig. 2*C*), suggesting that the protein degradation might be through 26S proteasome. In addition, the degradation of LUX mediated by phyA2 and phyA3 was further verified in tobacco. The presences of phyA3 and phyA2 severely reduced the abundance of LUX proteins (Fig. 2*D*), in agreement with the cell-free results (Fig. 2*C*). More interestingly, the protein enrichments of LUX were gradually enhanced when the protein concentrations of phyA3 and phyA2 were gradually decreased (*SI Appendix*, Fig. S6*B*). This phyA-mediated degradation of LUXs is unique because the presence of phyA3 and phyA2 does not result in the degradation of other circadian clock members such as J and E2 (homologs of GI) (*SI Appendix*, Fig. S6*C*). Collectively, these data showed that the phyA3 and phyA2 could interact with LUX and mediate its protein degradation.

### *PHYA2* and *PHYA3* Were Genetically Dependent on *LUX*.

**Fig. 3.**
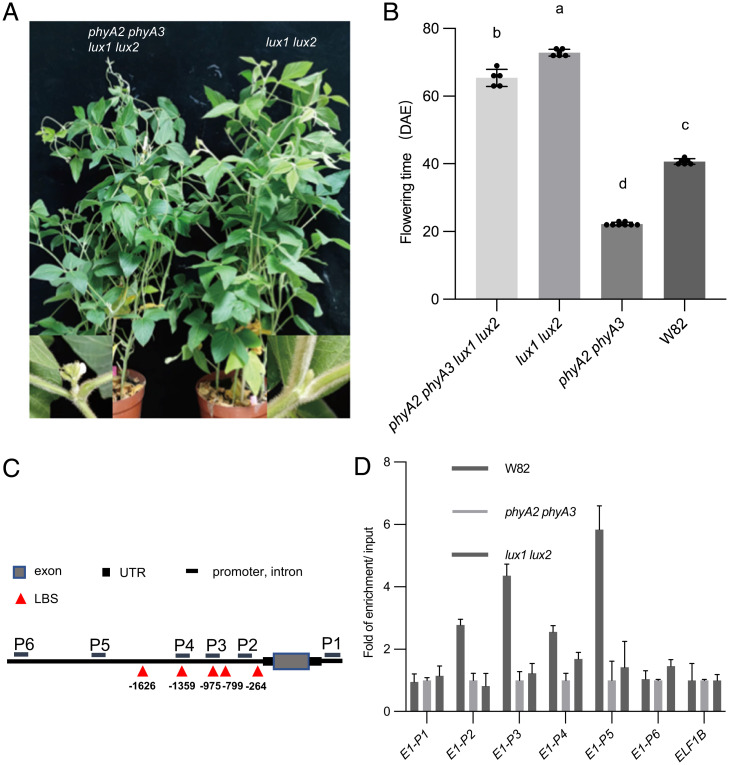
phyA2 and phyA3 are largely but not totally dependent on LUX. (*A*) Phenotypes of *lux1 lux2* and *phyA2 phyA3 lux1 lux2* mutants under LD (16-h light/8-h dark). (*B*) Flowering time of W82, *phyA2 phyA3*, *lux1 lux2*, *phyA2 phyA3 lux1 lux2* mutants under LD conditions (16-h light/8-h dark). Different letters indicate significant differences by Student’s *t* test (*P* < 0.05). The flowering time is shown as the mean values ± SD, *n* ≥ 5 plants. (*C*) Schematic of the *E1* gene and regions tested for enrichment in the ChIP assay. (*D*) ChIP of *E1* amplicons using W82, *phyA2 phyA3,* and *lux1 lux2* plants at Zeitgeber time 4. Native monoclonal antibody raised against phyA3 was used for ChIP assays.

Because LUXs are the key flowering enhancers that can directly bind to the promoter regions of *E1* and its homologs *E1la*, *E1lb* to repress their expression (30), and the expression of *E1*, *E1la*, and *E1lb* can be inhibited strongly in *phyA2 phyA3* plants (*SI Appendix*, Fig. S2). We then asked whether phyA2 and phyA3 mediated LUXs degradation could explain why *phyA2 phyA3* plants flowered early and down-regulated *E1* level under LD. To determine the genetic interaction between LUX and *PHYA2*, *PHYA3*, we generated quadruple mutant *phyA2 phyA3 lux1 lux2* by crossing between *phyA2 phyA3* and *lux1 lux2* double mutants [Guangzhou Mammoth (30)] and evaluated the flowering time under LD. The results showed that the flowering time of *phyA2 phyA3 lux1 lux2* is quite late, but is not as late as that of *lux1 lux2* (Fig. 3 *A* and *B*), which indicates that *PHYA2* and *PHYA3* are largely but not totally dependent on *LUX*.

To further explore whether the interaction of phyA2 and phyA3 with LUX caused the difference in transcription of *E1*, we performed chromatin immunoprecipitation (ChIP) experiments to confirm whether phyA3 is recruited to the promoter regions of *E1*. Leaves from 15 DAE *phyA2 phyA3* and W82, *lux1 lux2* plants grown under LD were harvested and subjected to ChIP-qPCR using phyA3 antibody. Our results showed that phyA3 can associate with *E1* promoters near the LBS (LUX binding sequence) motifs in wild-type W82 but failed in the *phyA2 phyA3* or *lux1 lux2* double mutants (Fig. 3 *C* and *D*), indicating that phyA3 was recruited to the promoter region of *E1* by LUX protein. Collectively, all these results suggest that phyA2 and phyA3 delay soybean flowering by suppression of transcription of *E1* family. This suppression is through the direct binding to the promoters of *E1* genes by the protein complex phyA-LUX in which phyA mediated the protein degradation of LUX to reduce the EC suppressions on *E1* gene family.

### phyA2 and phyA3 Physically Interacted with E1 and Its Homologs.

**Fig. 4.**
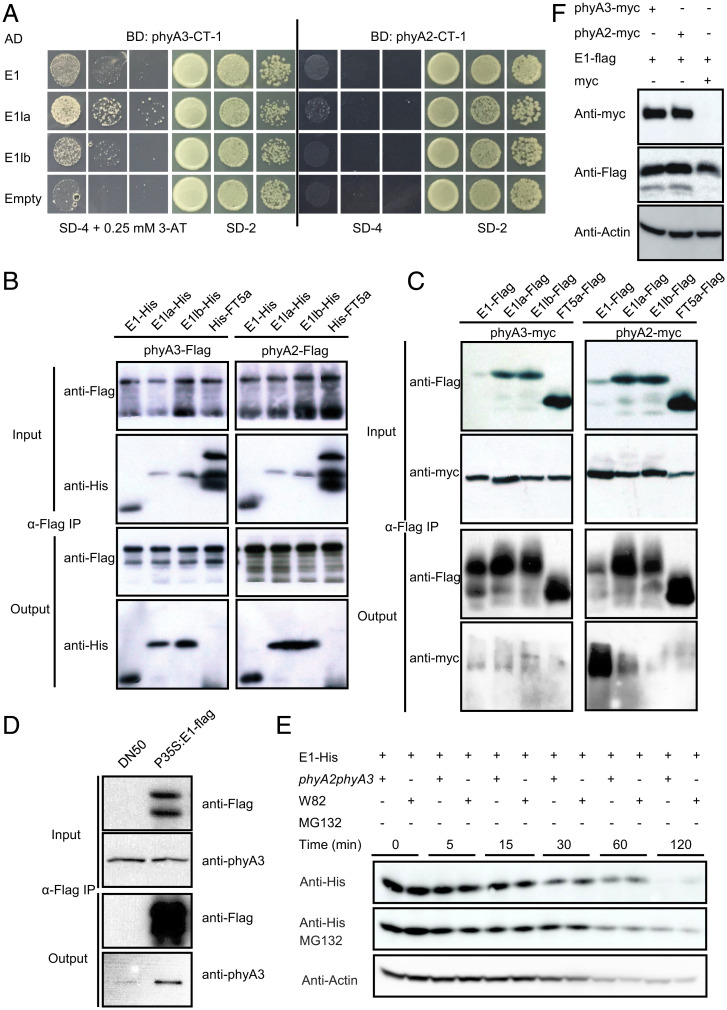
Protein interactions of E1 and its homologs with phyA2 and phyA3. (*A*) E1 and its homologs interact with phyA3-CT-1 (454 aa–1130 aa), but not with phyA2-CT-1 (447 aa–1123 aa) in yeast. Yeast cells transformed with indicated genes were selected on SD-2 (lacking Leu and Trp) and SD-4 (lacking Ade, His, Leu, and Trp) media with indicated 3-AT concentration. (*B*) phyA2 and phyA3 can pull down E1, E1la, E1lb. E1-His, E1la-His, E1lb-His, and His-FT5a proteins were expressed in *E. coli*, and phyA2-flag and phyA3-flag protein were expressed using an in vitro translation system. Purified proteins were used for the pull-down assay. phyA2-flag and phyA3-flag were detected with anti-flag antibody, and E1-His, E1la-His, E1lb-His, and His-FT5a protein were detected with anti-His antibody. (*C*) E1 and its homologs interact with phyA2 and phyA3 in *Nicotiana benthamiana* leaves in a co-IP assay. phyA2-myc and phyA3-myc were detected with anti-myc antibody, and E1-flag, E1la-flag, E1lb-flag, and FT5a-flag protein were detected with anti-flag antibody. (*D*) E1 interacts with phyA3 in soybean leaves in a co-IP assay. (*E*) Cell free in vitro degradation system indicating that E1-His is stabilized in protein extracts from W82 plants. Anti-actin was used as a sample loading control. (*F*) Transient expression in *N. benthamiana* leaves demonstrating that phyA2 and phyA3 stabilize E1 in plants. Anti-actin was used as a sample loading control.

From the Y2H screen, we also found that phyA2 and phyA3 interacted with E1la and E1lb. To further confirm the interactions between E1 family and phyA2, phyA3, we next used the C-terminal of phyA2 and phyA3 to conduct the Y2H assay. The C-terminal of phyA3 is interacted with E1, E1la, and E1lb, but the interactions of C-terminal of phyA2 with E1 family member were not detected (Fig. 4*A*). To verify these interactions between E1 family and phyA2, phyA3, we performed in vitro pull-down assay. Our data showed that phyA3-flag and phyA2-flag were able to pull down E1/E1la/E1lb-His, but not the control of His-FT5a (Fig. 4*B*). To detect if these interactions are light-dependent, we added chromophore phycocyanobilin to the pull-down system to allow the phytochromes to form Pr or Pfr forms under FR or R light treatment. We observed that E1 family protein could interact with both Pr and Pfr forms of phyA2 and phyA3 (*SI Appendix*, Fig. S7 *A* and *B*). In vivo interactions of E1/E1la/E1lb/FT5a-Flag and phyA2/phyA3-myc were evaluated by transient coexpression assay in tobacco leaves. phyA2/phyA3-myc was co-precipitated by the anti-flag antibody in the E1/E1la/E1lb-flag groups, but not in the control of FT5a-flag group, which further confirmed that E1 family interacted with phyA2 and phyA3 in planta (Fig. 4*C*). We also used the E1-flag overexpressing soybean plants (44) to conduct the co-IP assays to examine the in vivo association of E1 with phyA3. Our data showed that phyA3 also co-precipitated with E1-flag in soybean (Fig. 4*D*). Collectively, our data demonstrate that E1 family members physically interact with both phyA2 and phyA3 proteins.

**Fig. 5.**
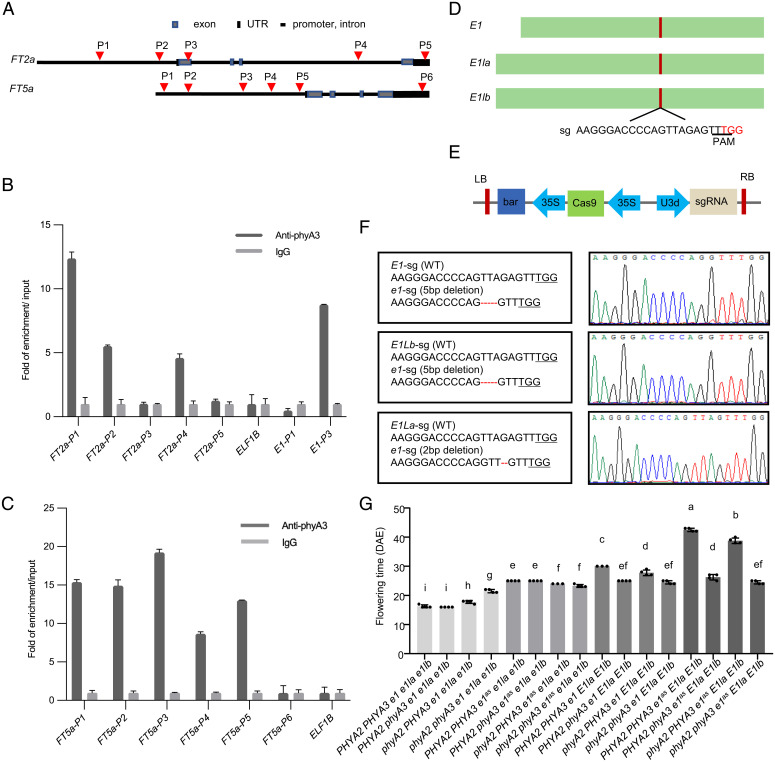
*PHYA2* and *PHYA3* are genetically dependent on *E1* and its homologs. (*A*) Schematic of the *FT2a* and *FT5a* gene and regions tested for enrichment in the ChIP assay. The red triangles indicate where the primers are. ChIP of *FT2a* and *E1* (positive control) (*B*) and *FT5a* (*C*) amplicons using ZK164 (*E1 E2 PHYA2 PHYA3*) genotype, Harosoy ecotype) plants. Native monoclonal antibody raised against phyA3 was used for ChIP assays. (*D*) The schematic of E1 family gene for CRISPR/Cas9 gene editing. The red bar indicates where the sgRNA is. (*E*) Schematic of CRISPR/Cas9 vector used for gene editing. (*F*) The 5-bp, 5-bp, and 2-bp deletion at target sites detected for *e1*, *e1la*, and *e1lb* by Sanger sequencing, respectively. (*G*) Flowering time of *PHYA2 PHYA3 e1 e1la e1lb*, *PHYA2 phyA3 e1 e1la e1lb*, *phyA2 PHYA3 e1 e1la e1lb*, *phyA2 phyA3 e1 e1la e1lb*, *PHYA2 PHYA3 e1^as^ e1la e1lb*, *PHYA2 phyA3 e1^as^ e1la e1lb*, *phyA2 PHYA3 e1^as^ e1la e1lb*, *phyA2 phyA3 e1^as^ e1la e1lb*, *PHYA2 PHYA3 e1 E1la E1lb*, *PHYA2 phyA3 e1 E1la E1lb*, *phyA2 PHYA3 e1 E1la E1lb*, *phyA2 phyA3 e1 E1la E1lb*, *PHYA2 PHYA3 e1^as^ E1la E1lb*, *PHYA2 phyA3 e1^as^ E1la E1lb*, *phyA2 PHYA3 e1^as^ E1la E1lb*, and *phyA2 phyA3 e1^as^ E1la E1lb* mutants under LD conditions (16-h light/8-h dark). Different letters indicate significant differences by Student’s *t* test (*P* < 0.05). The flowering time is shown as the mean values ± SD, *n* = 3 or 4 plants.

E1 is also a transcriptional factor that inhibit soybean photoperiodic flowering. Overexpressing E1 in DN50 background (despite multiple attempts, we failed to overexpress E1 in W82 background, probably because DN50 is easier to transform and get stable transformed plants) caused late flowering (*SI Appendix*, Fig. S8 *A* and *B*) (44). As a transcriptional factor, E1 can bind to the genomic region of *FT2a* and *FT5a* confirmed by ChIP-qPCR assay (*SI Appendix*, Fig. S8 *C–E*). Next, we then asked whether phyA2 and phyA3 regulate the protein stability of E1 through interacting with it. We used a cell-free system to test protein stability of E1 in the protein extracts of W82 and *phyA2 phyA3*. E1 is much more stable in buffer containing W82 plant extracts than those containing *phyA2 phyA3* plant extracts 15 min after E1 proteins were added into the degradation buffers (Fig. 4*E*). The presence of MG132 can slow down the rate of degradation of E1 in *phyA2 phyA3* extracts (Fig. 4*E*), which indicates that E1 may also degrade by 26S proteasome. To further confirm phyA2 and phyA3 could mediate E1 protein stabilization in plants, we transiently expressed LUX with or without phyA2/phyA3 in tobacco leaves. The results showed that with the presence of phyA2 and phyA3, E1 proteins are stabilized (Fig. 4*F*). To further explore the significance of interaction between phyA2 or phyA3 and E1, we performed ChIP experiment to check out whether phyA3 is recruited to the promoter regions of *FT2a* and *FT5a*, which are reported to be the downstream genes of E1. Leaves from 20 DAE ZK164 (*E1 E2 PHYA2 PHYA3*, Harosoy ecotype) plants grew under LD and were harvested and subjected to ChIP-qPCR using phyA3 antibody. The results showed that phyA3 physically associated with *FT2a* and *FT5a* promoters (Fig. 5 *A*–*C*). Taken together, these results indicate that phyA2 and phyA3 can form a complex with E1 and posttranscriptionally regulate the protein stability of E1 to enhance the suppression of *FT* genes to delay soybean flowering and maturity.

### The Genetic Interaction between *E1* Family and *PHYA2*, *PHYA3*.

To explore the genetic interactions between *E1* family and *PHYA2*, *PHYA3*, we first generated triple mutants of *e1 e1la e1lb* (Fig. 5 *D*–*F* and *SI Appendix*, Fig. S9) using CRISPR/Cas9 system, and then crossed *phyA2 phyA3* mutants with triple mutants of *e1 e1la e1lb*, of which the offspring generated 16 genetic combinations (Fig. 5*G*). We grew them under LD condition (R:FR ratio of 5:1) to observe their flowering times. To better compare the flowering difference, we divided them into four subgroups according to *E1* family genotypes. When *E1* family is normal (*e1^as^* allele in W82) or *e1* is null allele by knockout, *PHYA3* played a major and additive role with *PHYA2* in inhibiting flowering, in agreement with previous results under incandescent light (38, 39). Under *e1^as^ e1la e1lb* background, the role of *PHYA3* controlling flowering seemed to disappear. phyA2, however, had a minor role in flowering (Fig. 5*G* and *SI Appendix*, Fig. S10), indicating that *PHYA3* is genetically dependent on *E1la* and *E1lb* in this condition. Under *e1 e1la e1lb* background, *PHYA2 phyA3 e1 e1la e1lb*, *PHYA2 PHYA3 e1 e1la e1lb* flowered at the same time, indicating that *PHYA3* is genetically dependent on *E1* family. To our surprise, *phyA2 PHYA3 e1 e1la e1lb* flowered late compared with *PHYA2 phyA3 e1 e1la e1lb* and *PHYA2 PHYA3 e1 e1la e1lb*, and *phyA2 phyA3 e1 e1la e1lb* was the latest to flower in the *e1 e1la e1lb* group, and that is probably caused by the relatively slow vegetative growth of *phyA2 phyA3 e1 e1la e1lb*. Collectively, these data suggested that *PHYA3* and *PHYA2* are genetically dependent on *E1* family, with *PHYA3* predominantly dependent on *E1l* genes but *PHYA2* predominantly dependent on *E1* gene under this condition. All these results indicated that *PHYA2* and *PHYA3* genetically dependent on *E1* and its two homologs *E1la* and *E1lb*, which further supported their transcriptionally and posttranscriptionally regulations on E1 family to control photoperiod flowering and maturity, thus determines soybean adaptability and final yield productivity.

## Discussion

In *Arabidopsis*, phytochromes can be divided into two categories according to their stability under light, type I (light labile) and type II (light stable) (45). phyA, the receptor of FR light, is a light-liable type, which degrades rapidly under light, whereas phyB is light-stable, but still degrades slowly under R light (35, 46, 47). Phytochromes are very crucial regulators of flowering, and in many species such as model plants *Arabidopsis* and rice, phyB is the most important regulator among all phytochromes. phyA in these plants, however, are likely to be very sensitive to high proportions of R:FR light, and therefore their proteins cannot exist stably under such conditions. Nevertheless, under natural conditions, where sunlight is flooded with a very high proportion of FR light, especially in the early morning and dusk, the role of phyA in photoperiodic flowering under this condition may not be overlooked (43). On the other hand, in soybean, we proved that the function of phyB in regulating photoperiodic flowering is weak relative to that of phyA. After two rounds of genome replications, four copies of phyA are generated in soybean and phyA2 retains a function that is particularly sensitive to R light, similar to phyA in *Arabidopsis* (35). Intriguingly, the other copy produces a light stable form phyA3 that is particularly biochemically characteristic similar to *Arabidopsis* phyB, but not phyA (*SI Appendix*, Fig. S3). Moreover, this sensitivity to R light also perfectly explains the different responses of phyA2 and phyA3 to variations of ratios of R light to FR light in soybean (38, 39) (*SI Appendix*, Fig. S11). Therefore, we propose that the role of phyB in soybean photoperiodic flowering is so weak partially because phyA has evolved and generated neo-functions of phyA3 to replace a corresponding phyB-like function. It is worth noting that under the conditions of the incubator, the function of the phyA2 appears to be particularly weak because the fluorescent lamps, which are usually used in the incubators, contain a large proportion of R light. Under natural light conditions, however, there is more enriched FR light, and under close plant canopy conditions, where the proportion of FR light further increases, the role of phyA2 is indispensable, especially in high latitude regions (48). As phyA2 antibody recognizes both phyA2 and phyA1, we assume that phyA1 is also very sensitive to R light. Recent research indicates that phyA1 has a similar function as phyA2 in photoperiodic flowering under natural long days (49).

Both phyA and phyB can interact with ELF3 in *Arabidopsis*, although phyB and ELF3 do not appear to be involved in the same signaling pathway in regulating flowering (42), phyA and ELF3 antagonistically regulate *FT* expression levels (43). In rice (the other major SD plant system), the corresponding complex still exist (50). Therefore, although the functions of the phytochrome and EC complex have been reported to be antagonistic in many species, the mechanism by which they act is not fully understood. Our work initially revealed that phyA and J/ELF3 cannot form a complex in soybean, but demonstrated that phyA can form a complex with LUX and mediate the degradation of LUX, which is an important transcriptional factor directly binding to *E1*, thereby regulating flowering. CO is a core factor in photoperiodic flowering in many plants, such as *Arabidopsis* and rice (2). Compared with the importance of CO in these species, COL appears to have a relatively weak function in soybean, and instead what more important is another group of transcription factors: E1 and its homologous proteins (50). Together with what we previously reported on J and LUX (28, 30), we propose a photoperiodic flowering regulation model in soybean, namely phyA-LUX-E1-FT (*SI Appendix*, Fig. S11), which is different from the phyB-CO-FT flowering pathway in the LDP *Arabidopsis* and is also distinct from the SDP rice phyB-ELF3-Hd1-Hd3a flowering pathway (2, 51). Still, there are interesting common features with existing photoperiod pathways. In rice, the photoperiod response is mediated via phyB repressing ELF3 (14). Since LUX and ELF3 are both essential components of the EC, both rice and soybean have an identical network logic: light activates phytochrome, which represses EC, which then relieves expression of floral repressors, that ultimately represses *FT*. Thus, phytochrome proteins interact with and degrade EC to activate flowering repressors, such as E1 in soybean and Ghd7 and PRR37 in rice, resulting in dedayed flowering in both plants.

Although soybean possess a unique, E1-centered regulatory network for photoperiodic flowering (19), previous reports on *E1* mostly exist in the regulation of its transcriptional level, and little is known about posttranscriptional regulation of E1. Our work reports that phyA can form a transcriptional complex with E1 to regulate the transcription of the downstream *FT2a* and *FT5a* genes, which means the regulation of phyA on E1 is multilayered. This is how phyA double checks the soybean photoperiodic flowering pathway: phyAs not only regulates the transcriptional level of *E1* by binding to LUX protein, but also regulates its posttranscriptional level by directly binding to E1 protein (*SI Appendix*, Fig. S11). Nevertheless, genetic analysis showed that the flowering time of *phyA2 phyA3 lux1 lux2* and *lux1 lux2* differed by only 1 wk, indicating that phyA2 and phyA3 regulate E1 family mainly at the transcriptional level rather than the posttranscriptional level. Until now, we have not known how phyA can simultaneously degrade LUX but stabilize E1 in soybean, which may be related to other proteins bound by phyA. phyA is often involved in sophisticated large complexes, regulating multiple signaling pathways by stabilizing and degrading other proteins. For example, in *Arabidopsis*, phyA can regulate photoperiod flowering by stabilizing CO (3), and it can also regulate photomorphogenesis by degrading PIF3 (52, 53). However, the mechanisms of these are still not particularly clear (53).

Because of the importance of phyA, LUX, J, and E1 and its homologs in the photoperiodic flowering pathway, they all have numerous allelic variations in nature, and these abundant genetic resources will in turn allow us to precisely manipulate soybean photoperiodic flowering through molecular design breeding. The detailed dissection of the photoperiod flowering pathway is not only a matter of adjusting flowering time of crops, but also an important passport for regulating plant yield and fitness. The establishment of the phyA-LUX-E1-FT regulatory pathway is different from any known photoperiodic flowering regulatory pathway in other plants or crops. Therefore, it also lays a foundation that photoperiodic flowering pathways are divergent in different plant species.

## Materials and Methods

### Plant Materials, Growth Conditions, and Phenotyping.

In this study, the soybean (*Glycine max* [L.] Merr.) cultivar Williams 82 (W82) was used as the wild type. Plants for expression analysis, ChIP assay, Western blot, and transcriptome analysis were grown under long day conditions (LD, 16 h light/8 h dark) in a plant growth chamber with temperature at 25 °C. Flowering time was recorded at the R1 stage (days from emergence to the first open flower appeared at any node on the main stem).

### Quantitative RT-PCR.

Total RNA was extracted from youngest fully expanded trifoliate leaves at 20 DAE using RNApure Plant Kit (CWBIO). The RNA was reverse transcribed to cDNA with M-MLV reverse transcriptase kit (Takara). Quantitative RT-PCR (qRT-PCR) was performed on a Roche LightCycler 480 system (Roche) using 2× Ultra SYBR Green qPCR Mix (CISTRO). *Tubulin* was used as an internal control. The primers are listed in Datasets S1.

### Generation of Antibody to phyA2 and phyA3 and Immunoblotting.

Antibodies were generated in mouse (made by Beijing Protein Innovation) against a phyA3- and phyA2-specific peptide, corresponding to last 300 amino acids of phyA3 and phyA2. The recombinant proteins used were expressed in *Escherichia coli* and purified. The specificity of the antibodies to phyA3 and phyA2 were tested by immunoblotting and are shown in *SI Appendix*, Fig. S3. The use of experimental animals was approved by the Science and Technology Committee of Shanghai, China.

### Transient Expression.

Agrobacterium (strain GV3101) bacteria containing indicated constructs with corresponding concentration were coinfiltrated into young but fully expanded leaves of the tobacco using a needleless syringe. After infiltration, plants were grown under dark for 1 d and 16-h-light/8-h-dark for 1d. Then the leaves were harvested with liquid nitrogen and for protein extraction with protein extraction buffer (50 mM Tris–HCl pH 7.5, 150 mM NaCl, 5 mM EDTA, 0.1% Triton X-100, and protease inhibitor mixture). Corresponding volume of 5× SDS loading buffer were added and boiled for 5 min. After centrifugation at 12,000 × *g* for 1 min at 22 °C, the supernatant was subjected to immunoblotting analysis and immunoblotted as described in *SI Appendix*.

### Yeast Two Hybrid Assays.

The detailed transformation procedure was described in *SI Appendix*. The yeast cells were grown on a minimal medium SD/-Leu-Trp according to the manufacturer’s instructions (Clontech). Positive clones were selected on SD/-His-Leu-Trp or SD/-His-Leu-Trp-Ade selection medium with extra 3-amino-1,2,4-triazole.

### Co-IP.

After infiltration, plants were grown under dark for 1 d and 16-h-light/8-h-dark for 2 d. Then the leaves were harvested with liquid nitrogen and for protein extraction with protein extraction buffer (50 mM Tris –HCl pH7.5, 150 mM NaCl, 5 mM EDTA, 0.1% Triton X-100, and protease inhibitor mixture). Flag beads (Sigma) were washed for three times with protein extraction buffer before they were added to extracted protein. The tubes were rotated for protein binding at 4 °C for 2 h and the beads were washed five times with protein extraction buffer and 60 μL of 2× SDS loading buffer were added and boiled for 5 min. After centrifugation at 12,000 × *g* for 1 min at 22 °C, the supernatant was subjected to immunoblotting analysis. The antibody anti-myc and anti-flag were from Sigma.

### In Vitro Pull Down Assay.

The detailed proteins preparation procedure was described in *SI Appendix*. The in vitro translated PHYA3 or PHYA2 proteins together with 3 μg myelin basic protein (MBP)/His purified proteins were diluted with pull down buffer (50 mM Tris HCl [pH 7.5], 100 mM NaCl, 2 mM EDTA, 1% dimethyl sulfoxide [DMSO], 2 mM dithiothreitol [DTT], 0.1% Nonidet P-40, 1 μM phenylmethylsulfonyl fluoride [PMSF], 1× mixture [Roche]). Flag beads/MBP beads were washed with pull down buffer and then added to the protein mix and they were incubated for 2 h under indicated conditions. Then, the beads were washed five times with pull down buffer. Bound proteins were eluted by boiling in 2× SDS loading.

### ChIP Assay.

Leaf samples were collected from 15-d-old plants at Zeitgeber time 4 under LD conditions from *W82*, *phyA2 phyA3*, *lux1lux2* mutant plants in Fig. 3*D*. Leaf samples were collected from 20-d-old plants at Zeitgeber time 4 under LD conditions from ZK164 plants in Fig. 5 *B* and *C*. ChIP experiment was performed as previously described (54). Samples were grounded in liquid nitrogen and ChIP extraction buffer I was added, then formaldehyde was added to the final concentration to1% and fixed in 4 °C for 10 min. Nuclei were isolated and sonicated as previously described (54). The soluble chromatin was immunoprecipitated by antibody to E3 with protein A/G beads (Bio-Rad: 161-4023). The immunoprecipitated DNA was recovered and analyzed by quantitative RT-PCR in triplicate. Relative fold enrichment was calculated by normalizing the amount of a target DNA fragment against that of a genomic fragment of a reference gene, *ELONGATION FACTOR 1 GmELF1B* (Glyma.02G276600.1) and then by normalizing the value of input DNA. The primers used for amplification are listed in Dataset S1.

### Cell-Free In Vitro Degradation Assay.

The cell-free protein degradation assay was performed as described previously with some modifications (55). Leaf samples were collected from 20-d-old plants at Zeitgeber time 0, 4, 8, 12, 16, 20, and 24 under LD conditions from W82, *phyA2 phyA3* mutant plants. The samples of different time periods were mixed together, and the protein was extracted with cell degradation buffer (25 mM Tris–HCl pH 7.5, 10 mM NaCl, 10 mM MgCl_2_, 5 mM DTT, 1 mM PMSF, 10 mM ATP, and 100 μM cycloheximide). The ones to which that MG132 was added, the final concentration of MG132 was 40 μM. Then an equal amount of prokaryotic-expressed LUX1-MBP, LUX2-MBP, or E1-His was added to *phyA2 phyA3* or W82 extracts for time course degradation assay at room temperature.

## Supplementary Material

Supplementary File

Supplementary File

## Data Availability

All study data are included in the article and/or supporting information.
