# Supplementary material for: Novel and multifaceted regulations of photoperiodic flowering by phytochrome A in soybean
Source: Proc Natl Acad Sci U S A. 2022 Oct 3;119(41):e2208708119. doi: 10.1073/pnas.2208708119 (PMC9565047; doi:10.1073/pnas.2208708119)
Supplement: Supplementary File [file pnas.2208708119.sapp.pdf]

Supplementary Information for
Novel and multifaceted regulations of photoperiodic flowering by phytochrome A in soybean

Xiaoya Lin[a,1,2], Lidong Dong[a,1], Yang Tang[a,1], Haiyang Li[a,c,1], Qun Cheng[a], Hong Li[d], Ting Zhang[a], Lixin Ma[a], Hongli Xiang[a], Linnan Chen[a], Haiyang Nan[a], Chao Fang[a], Sijia Lu[a], Jigang Li[d], Baohui Liu[a,b,2], Fanjiang Kong[a,b,2]

[1] X.L., L.D., Y.T. and H.L. contributed equally to this work.

[2] To whom correspondence may be addressed.
Email: F.K. (kongfj@gzhu.edu.cn), B.L. (liubh@gzhu.edu.cn) or X.L. (xiaoyalin@gzhu.edu.cn)

This PDF file includes:

Supplementary text
Figures S1 to S11
Legends for Datasets S1
SI References

***SI Appendix* Materials and Methods**

Plasmid construction and plant transformation

The CDS (started with ATG, ended before stop codon) of *PHYA3* and *PHYA2* were amplified from Williams 82 de-etiolated seedlings by KOD plus neo. The pYLCRISPR–Cas9P35S-BS, which constructed before, was used for making the vector to knockout *PHYA3* and *PHYA2*. The target sequence adapters for *PHYA3* and *PHYA2* were designed by CRISPR-GE (http://skl.scau.edu.cn/). Four gene targets were selected and integrated into the vector according to the protocol previously reported. The obtained CRISPR-Cas9 vectors was transformed to EHA101 strains and then transformed to soybean accession Williams 82 as previously reported. Agrobacterium-mediated transformation was performed as described previously (1). All the primers used for vector construction are listed in Datasets S1.

Western blot

To analyze the protein accumulation in different plants, total proteins of W82 and *phyA2 phyA3* from different time and different conditions were extracted with protein extraction buffer (50mM Tris –HCl pH7.5, 150mM NaCl, 5mM EDTA, 0.1% Triton X-100 and protease inhibitor cocktail). The homogenate was clarified by centrifugation at 14,000 g for 10 min at 4°C, and aliquots of the supernatant were combined with 2 × SDS sample loading buffer and heated at 95°C to for 5 min to denature the protein. The antibody anti-Flag and myc was obtained from Sigma.

Yeast two hybrid assays

The yeast two-hybrid assay was performed as previously described (2). In brief, the full-length CDS of *PHYA2*, *PHYA3* were introduced into D153 plasmids (3), the C terminal of *PHYA3* (454aa-1130aa)) and *PHYA2* (47aa-1123aa) were introduced to pGBKT7 vector, and the full length CDS of E1, E1la, E1lb, LUX1, LUX2 were introduced to pGADT7, and then the indicated plasmids were cotransformed into yeast strain Y2H Gold (Clontech). The assay also screened the interactions of COP1a, COP1b, COL4, COL1a, COL2a, COL2b, COL7a, COL7b, E2, LHY1b, LHY2b to phyA and showed negative results. For J and phyA3-CT (626aa-1130aa), phyA2-CT (619aa-1123aa), J was introduced to pGBKT7 vector, and phyA3-CT, phyA2-CT were introduced to pGADT7, and then the indicated plasmids were cotransformed into yeast strain Y2H Gold (Clontech).

Co-immunoprecipitation (Co-IP)

Co-IP assays were performed as previously described (4). Plant expression vectors for phyA3-myc and phyA2-myc expression vectors were made by LR reactions using pEntry-*PHYA3*/*PHYA2* and pK7MYCWG2. Briefly phyA3-myc, phyA2-myc, E1-3flag, E1la-3flag, E1lb-3flag, LUX1-3flag, LUX2-3flag, FT5a-flag were transformed into Agrobacterium strain GV3101, and different combinations were mixed for injection into *Nicotiana benthamiana* leaves.

***In-vitro* pull down assay**

The full-length CDS of *PHYA3* and *PHYA2* fused with 3flag were cloned into the pMAL-c5X vector. MBP, MBP-LUX1 and MBP-LUX2 were purified with amylose resin (NEB). His-E1, His-E1la, His- E1lb, FT5a-His were purified with Ni-NTA (QIAGEN). phyA3-3flag and phyA2-3flag protein was purified using TnT Quick Coupled Transcription/Translation Systems according to the manufacturer's instructions (Promega, L1170). Holoproteins of PHYA3 and PHYA2 were generated by incubating the respective apoproteins with 20 mM phycocyanobilin (PCB) for 1 h in the dark on ice to allow the incorporation of the chromophore (J&K Scientific, P14137) like previously reported (5).

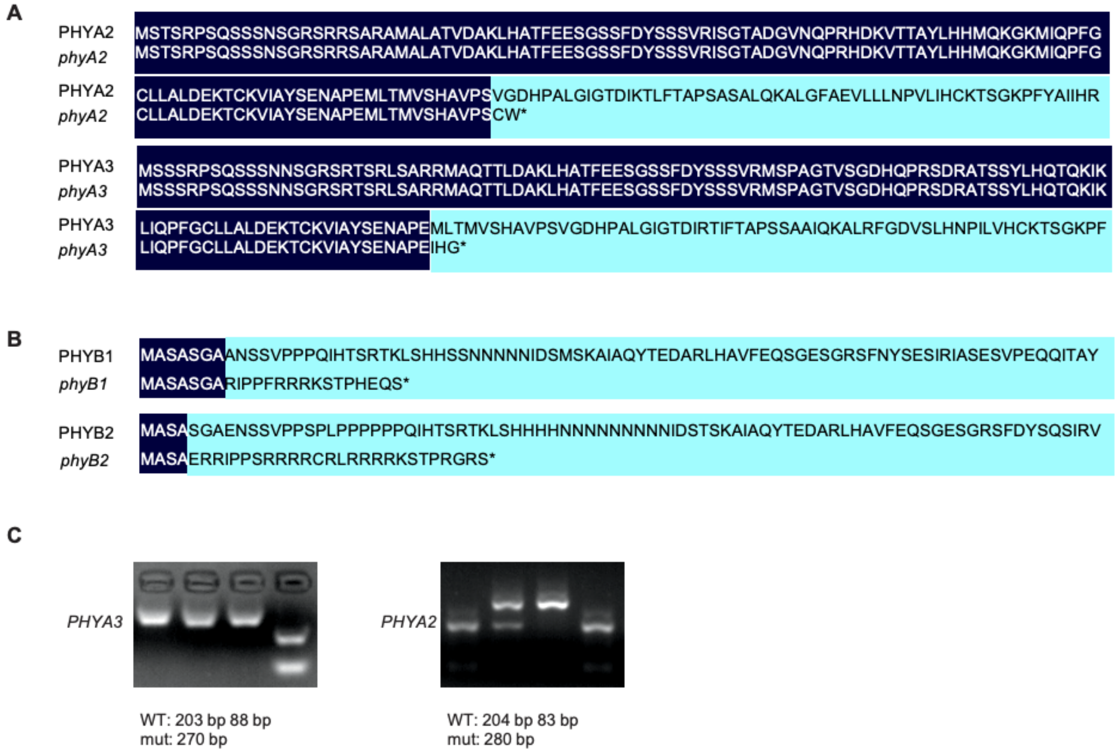

Fig. S1. Identification of *phyA2 phyA3 and phyB1 phyB2* mutants by CRISPR/Cas9 in soybean. (A) Protein sequences of *phyA2* and *phyA3* after gene editing. (B) Protein sequences of *phyB1*and *phyB2* after gene editing. (C) CAPs method for identifying *phyA3* and *phyA2* mutants.

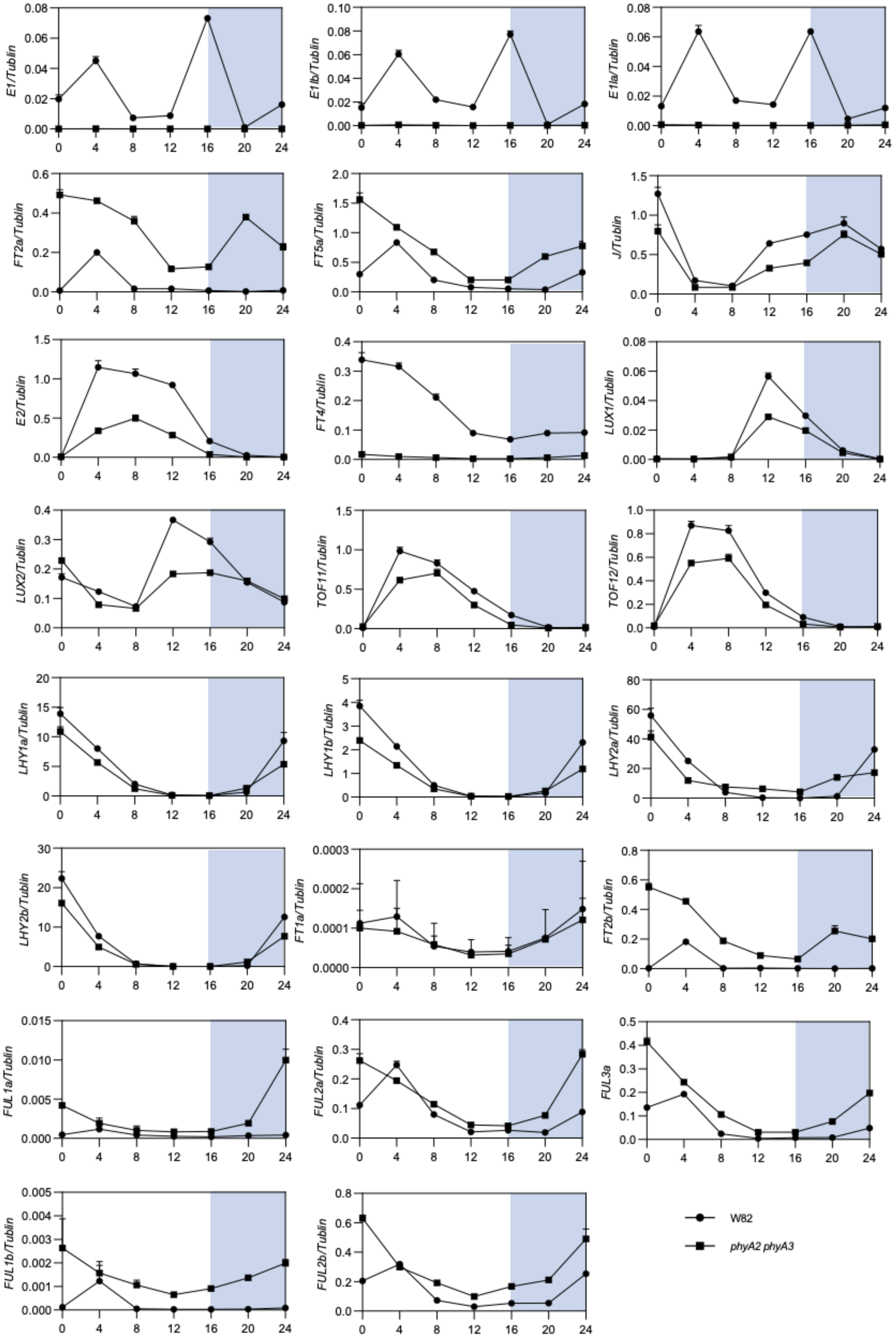
E1/Tublin
E1lb/Tublin
E1la/Tublin
FT2a/Tublin
FT5a/Tublin
J/Tublin
E2/Tublin
FT4/Tublin
LUX1/Tublin
LUX2/Tublin
TOF11/Tublin
TOF12/Tublin
LHY1a/Tublin
LHY1b/Tublin
LHY2a/Tublin
LHY2b/Tublin
FT1a/Tublin
FT2b/Tublin
FUL1a/Tublin
FUL2a/Tublin
FUL3a
FUL1b/Tublin
FUL2b/Tublin
W82
phyA2 phyA3

Fig. S2. Temporal expressions of the flowering genes in W82 and *phyA2 phyA3* mutants in LD at 20 DAE. Time-course expression of *E1*, *E1la*, *E1lb*, *FT2a*, *FT5a*, *FT4*, *FT1a*, *FUL1a*, *FUL1b*, *FUL2a*, *FUL2b*, *FT2b*, *FUL3a*, *J*, *E2, LUX2*, *LUX1*, *TOF11*, *TOF12*, *LHY1a*, *LHY1b*, *LHY2a*, *LHY2b* in W82 and *phyA2 phyA3* plants. Data shown relative to the control gene *Tubulin* and represent means ± SD for three biological replicates.

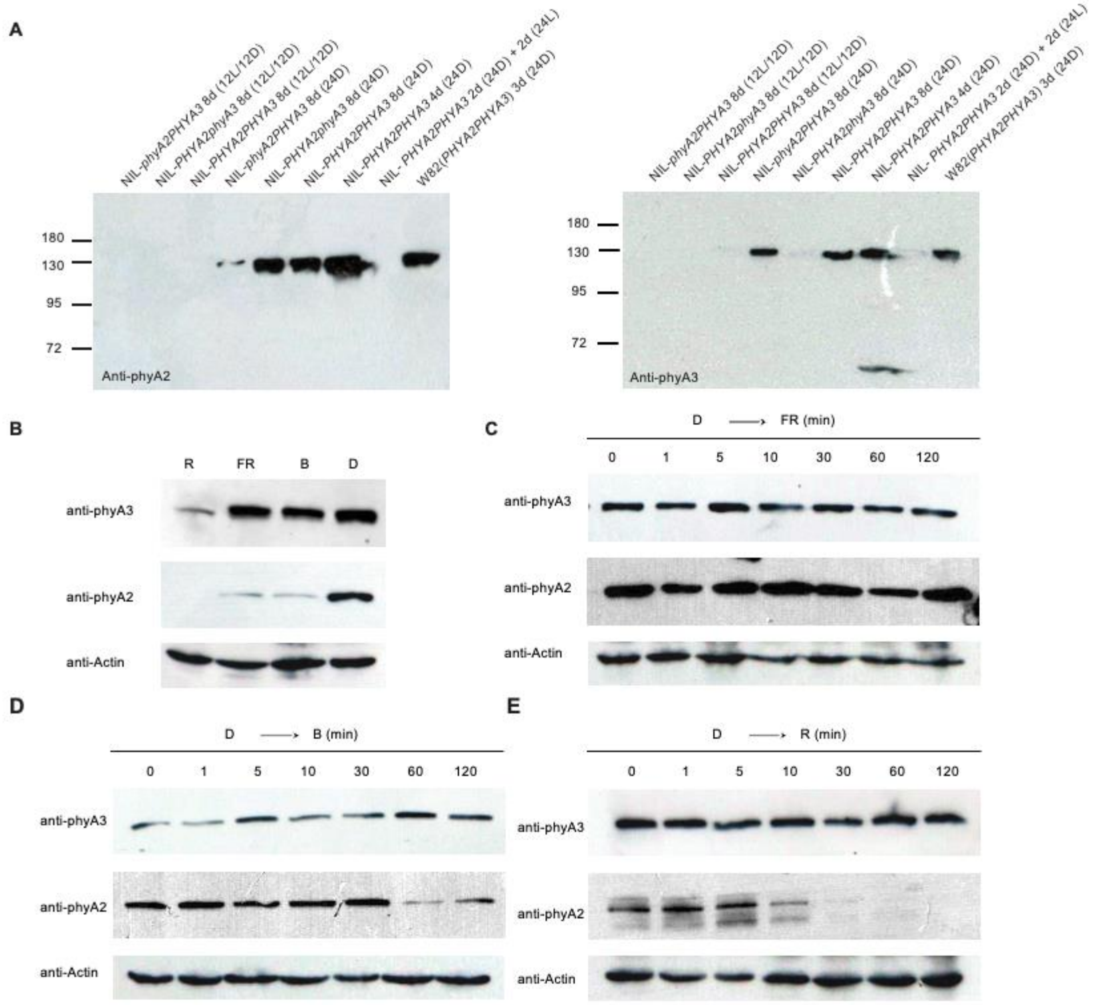

Fig. S3. phyA2 and phyA3 Proteins are differentially regulated in different light conditions. (A) Total protein of indicated seedlings grown under listed conditions were tested by immunoblot with phyA2 and phyA3 antibody. (B) Immunoblots showing phyA3 and phyA2 protein levels in 3-d-old W82 seedlings grown in D or continuous FR, R, B light. (C to E) Immunoblots showing that phyA3 and phyA2 proteins accumulated in different patterns after the 4-d-old etiolated W82 seedlings were transferred to FR (B), B (C), or FR (D) light conditions for the indicated time periods. Anti-Actin was used as a sample loading control.

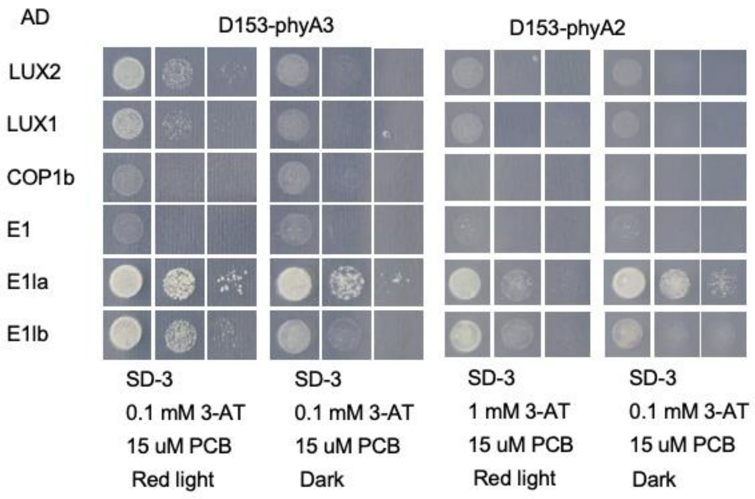

Fig. S4. the interactions of phyA with LUX and E1ls are light–independent. Yeast two-hybrid assays showing that the interactions of phyA2 and phyA3 to LUX1, LUX2, E1la and E1lb are not light dependent. The Pfr and Pr form exist under red light and dark, respectively.

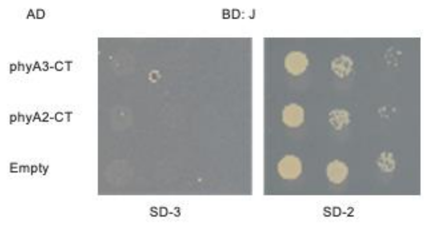

Fig. S5. J did not interact with phyA2 and phyA3 in yeast. Yeast two hybrid results with J-BD bait constructs and the C-terminal phyA3 (phyA3-CT: 626aa-1130aa), C-terminal phyA2 (phyA2-CT: 619aa-1123aa) prey plasmid.

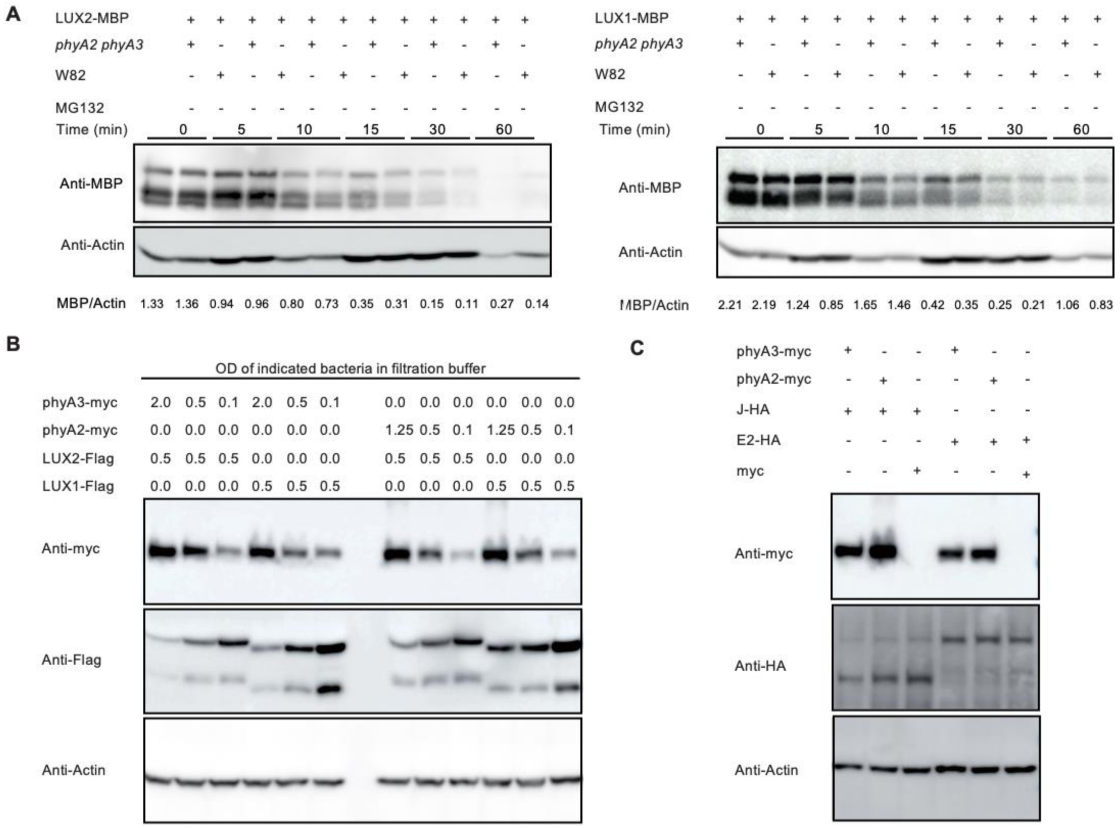

Fig. S6. phyA2 and phyA3 mediated the degradation of LUX, and did not influence protein stability of J and E2. (A) Cell free in-vitro degradation system indicating that LUX1/ 2-MBP is stabilized in protein extracts from *phyA2 phyA3* plants. Anti-Actin was used as a sample loading control. Relative band intensities were quantified and normalized by image J. (B) Transient transformation experiments in tobacco leaves demonstrated that the accumulation of LUX1 and LUX2 gradually decreased with the accumulation of phyA2 and phyA2 proteins. (C) Transient transformation experiments proved that the presence or absence of phyA2 and phyA3 did not affect the protein accumulation of J and E2. Anti-Actin was used as a sample loading control.

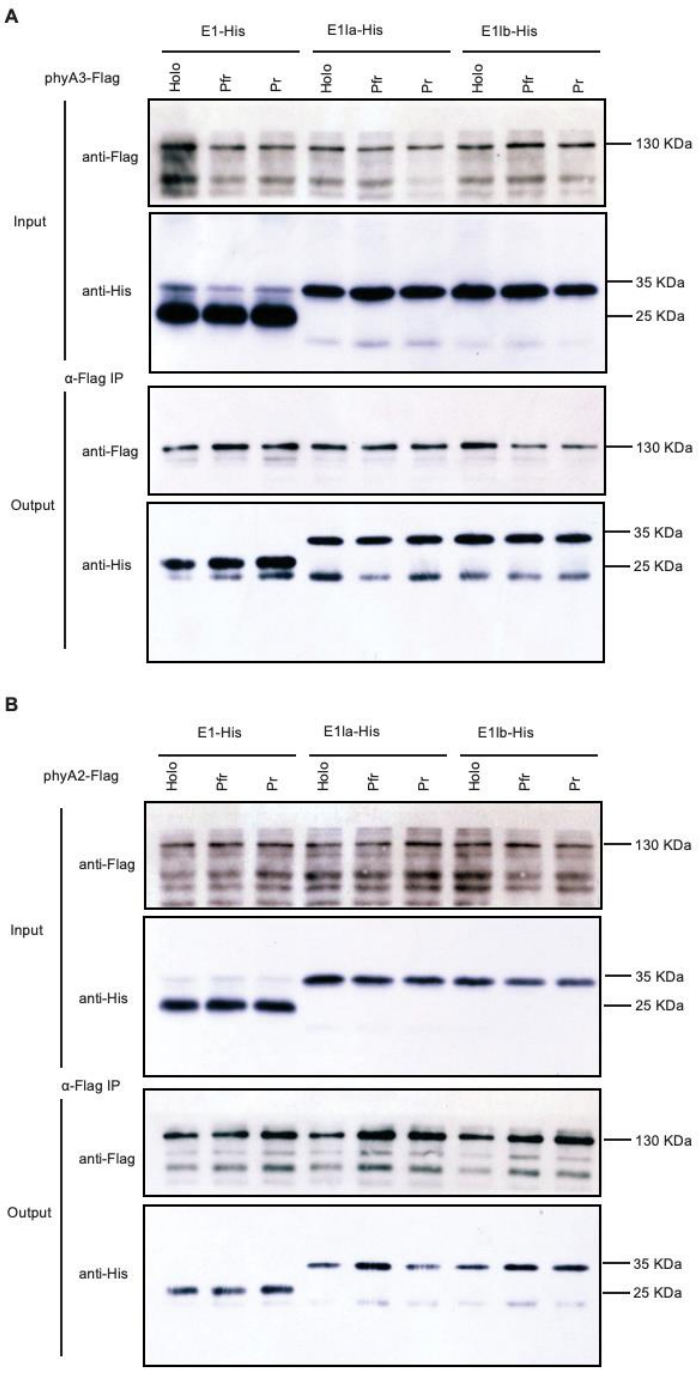

Fig. S7. E1 and its homolog interacted with phyA2 and phyA3 in a light independent manner. Pull-down assays using either in vitro translated phyA3-flag (A) or phyA2-flag (B) proteins to pull down E1, E1la, E1lb. The pull down assays were carried out with the phy haloprotein (Halo, dark) or the holoprotein in either the Pfr (R light) or Pr (FR light) form as indicated.

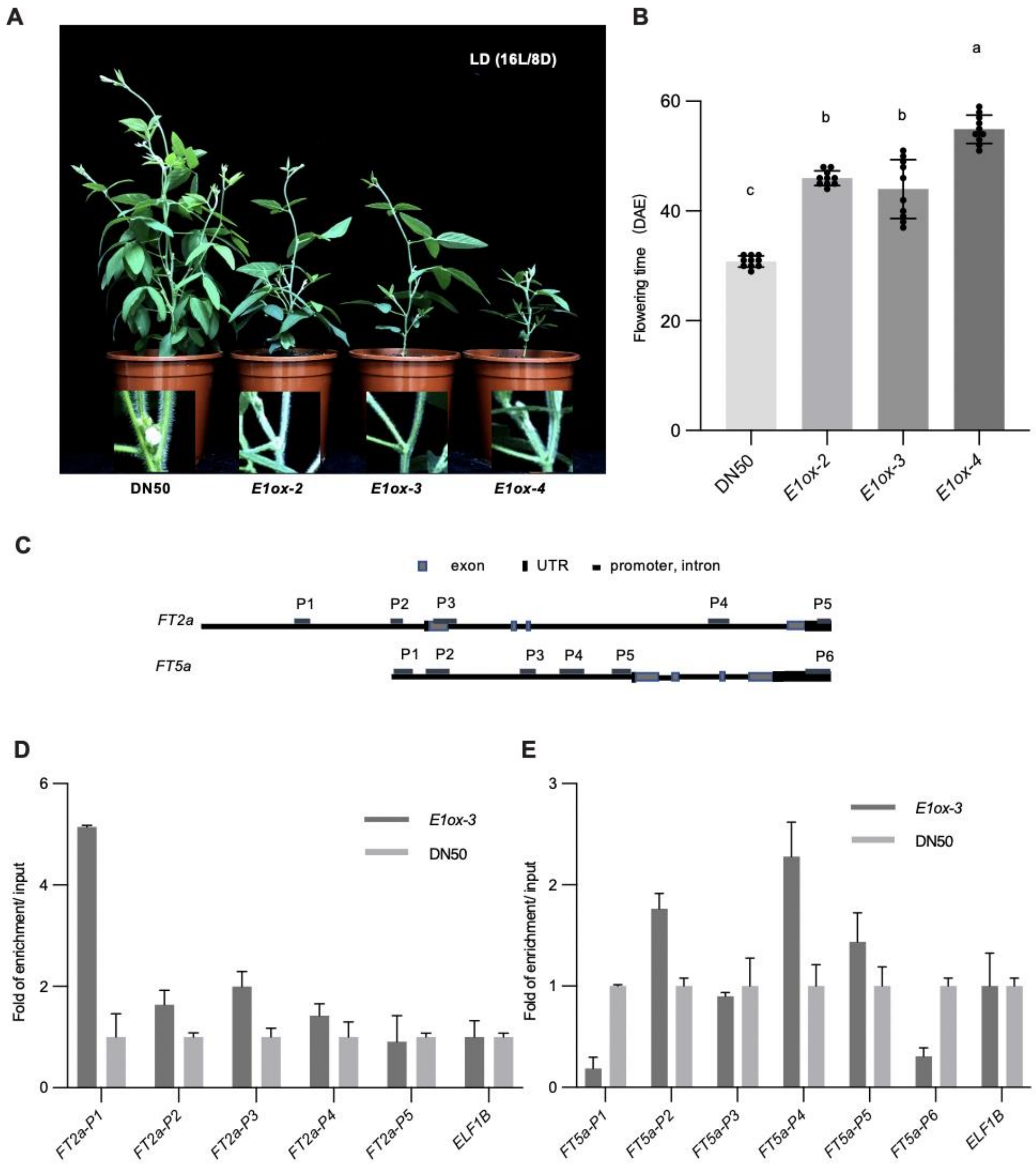

Fig. S8. E1 is a flowering inhibitor. (A-B) Phenotypes of wild-type plants (WT, DN50) and *E1 overexpressing* (*ox*) plants under LD (16-h light/ 8-h dark). Different letters indicate significant differences by Student test ($P < 0.05$). (C) Schematic of the *FT2a* and *FT5a* gene and regions tested for enrichment in the ChIP assay. ChIP of *FT2a* and *E1* (positive control) (D) and *FT5a* (F) amplicons using *E1ox-3* and DN50 plants. Flag antibody was used for ChIP assays.

E1	MSNPSDEREQCQKKTKSTICEASNFRTSRRRFCSNNKNEEEMNNKGVSTTLKLYDDPWKIKKTLTDSDLGILSRLLLAADLVKKQILPML
e1	MSNPSDEREQCQKKTKSTICEASNFRTSRRRFCSNNKNEEEMNNKGVSTTLKLYDDPWKIKKTLTDSDLGILSRLLLAADLVKKQILPML
E1	GAYHARAAETVRVWDMDTKSMHQLVLKRWSSSKSYVLIGKWNQDFVRRRDLRKGDEIGFHWDPYNCVFNFCVLKQAMPE
e1	GAYHARAAETGLGHGHQIHAPTRSKAMVFIQELCSYWKVEPRFRQKKRSQER*
E1la	MSLQSQIPSKFKIIPISSNMSNPSDEKEQCQKKRKSTICEASNFKTSRRRFFSNKNEEDMNKGVSTTLKLYDDPWKIKKTLTDSDLGILS
e1la	MSLQSQIPSKFKIIFISSNMSNPSDEKEQCQKKRKSTICEASNFKTSRRRFFSNKNEEDMNKGVSTTLKLYDDPWKIKKTLTDSDLGILS
E1la	RLSLAADLVKKQILPMLGADHARAAETEEGTPVRVWDMDKSMHQLVLKRWSSSKSYVLIGKWNQDFVRRRDLKKGDEIGFHWDPYN
e1la	RLSLAADLVKKQILPMLGADHARAAETEEGTPGLGHGHQIHAPARSKAMV FIQELCSYWKVEPRFREKKR SQERG*
E1la	CVFNFCVLKRAMPEN
e1la	
E1lb	MSLQSQIPSKFKIIPISSNMSNHSDEKEQCQKKRKSTICEASNFRTSRRRFCSNKNEEEMNKGVSTTLKLYDDPWKIKKTLTDSDLGILS
e1lb	MSLQSQIPSKFKIIPISSNMSNHSDEKEQCQKKRKSTICEASNFRTSRRRFCSNKNEEEMNKGVSTTLKLYDDPWKIKKTLTDSDLGILS
E1lb	RLSLATDLVKKQILPMLGADHARAAETEEGTPVRVWDMDTKSMHQLVLKRWSSSKSYVLIAKWNQDFVRRRDLKKGDEIGFHWDPYN
e1lb	RLSLATDLVKKQILPMLGADHARAAETEEGTPVSLGHGHQIHAPARSKAMVFFQELCSYCKVEPRFREKKRPQERG*
E1lb	CVFNFCVLKRAMPEN
e1lb	

Fig. S9. Generation of *e1 e1la e1lb* mutants by CRISPR/Cas9 in soybean. Protein sequences of *e1*, *e1la* and *e1lb* after gene editing.

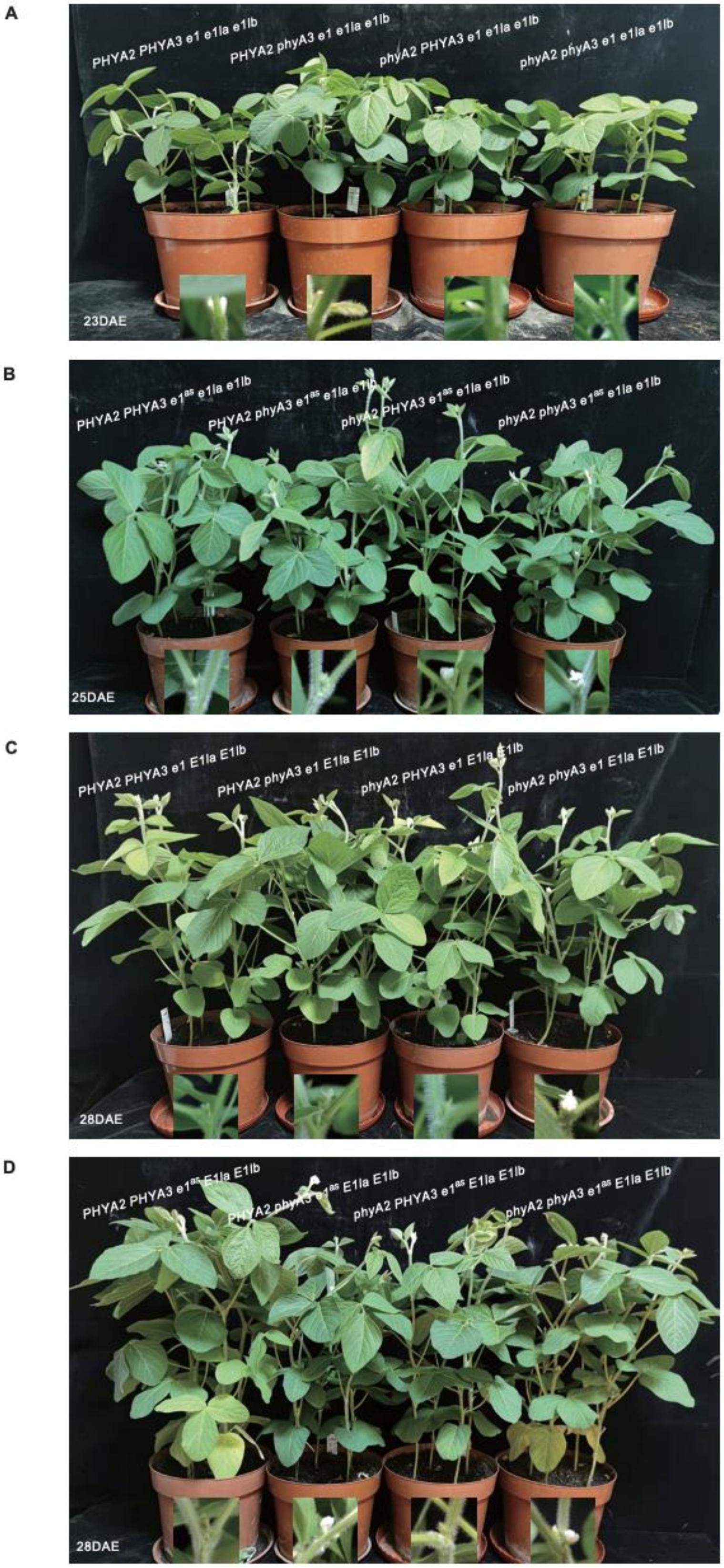
A
PHYA2 PHYA3 e1 e1la e1lb
PHYA2 phyA3 e1 e1la e1lb
phyA2 PHYA3 e1 e1la e1lb
phyA2 phyA3 e1 e1la e1lb
23DAE
B
PHYA2 PHYA3 $e1^{as}$ e1la e1lb
PHYA2 phyA3 $e1^{as}$ e1la e1lb
phyA2 PHYA3 $e1^{as}$ e1la e1lb
phyA2 phyA3 $e1^{as}$ e1la e1lb
25DAE
C
PHYA2 PHYA3 e1 E1la E1lb
PHYA2 phyA3 e1 E1la E1lb
phyA2 PHYA3 e1 E1la E1lb
phyA2 phyA3 e1 E1la E1lb
28DAE
D
PHYA2 PHYA3 $e1^{as}$ E1la E1lb
phyA2 PHYA3 $e1^{as}$ E1la E1lb
phyA2 phyA3 $e1^{as}$ E1la E1lb
28DAE

Fig. S10. *PHYA2* and *PHYA3* are genetic dependent on *E1* family genes. Phenotypes of *PHYA2 PHYA3 e1 e1la e1lb*, *PHYA2 phyA3 e1 e1la e1lb*, *phyA2 PHYA3 e1 e1la e1lb*, *phyA2 phyA3 e1 e1la e1lb* (A), *PHYA2 PHYA3* $e1^{as}$ *e1la e1lb*, *PHYA2 phyA3* $e1^{as}$ *e1la e1lb*, *phyA2 PHYA3* $e1^{as}$ *e1la e1lb*, *phyA2 phyA3* $e1^{as}$ *e1la e1lb* (B), *PHYA2 PHYA3 e1 E1la E1lb*, *PHYA2 phyA3 e1 E1la E1lb*, *phyA2 PHYA3e1 E1la E1lb*, *phyA2 phyA3e1 E1la E1lb* (C), *PHYA2 PHYA3* $e1^{as}$ *E1la E1lb*, *PHYA2 phyA3* $e1^{as}$ *E1la E1lb*, *phyA2 PHYA3* $e1^{as}$ *E1la E1lb*, *phyA2 phyA3* $e1^{as}$ *E1la E1lb* (D) under LD (16-h light/8-h dark) at indicated time period.

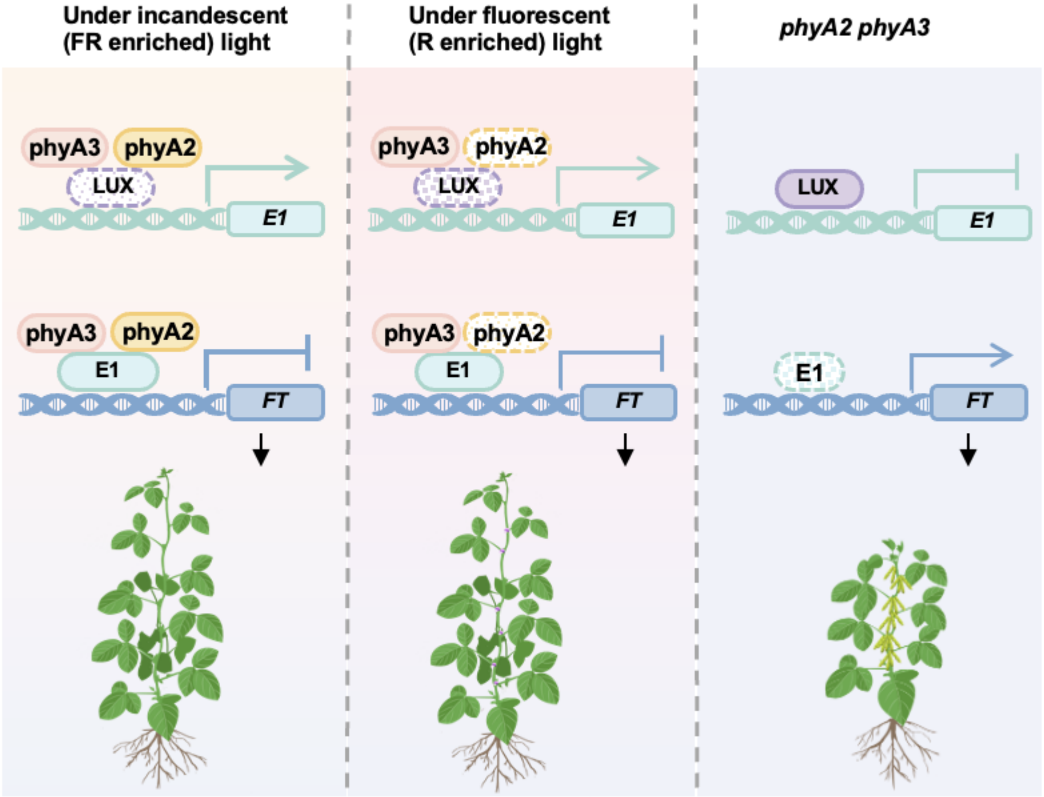

Fig. S11. Working model for phyA2 and phyA3 to regulate soybean photoperiod flowering. phyA2 and phyA3 have different protein stability under different light. When there is much R light, only phyA3 can remain stable and function. When there is more FR light, both phyA3 and phyA2 proteins remain stable and function together. Under fluorescent light (R-light enriched) conditions, phyA2 protein is down-regulated, so only phyA3 protein exists. On the one hand, phyA3 proteins can bind to LUX proteins and down-regulate them. Releasing the inhibition of LUX on *E1*, which allows *E1* to be transcribed and translated in large quantities. On the other hand, phyA3 can also interact with E1 protein to stabilize E1 protein, so that the expressions of downstream *FT2a* and *FT5a* genes are inhibited, thereby inhibiting flowering. When LUXs are not present, the inhibition of LUX to *E1* is released. The large amount of transcribed the translated E1 is stabilized by phyA3, leading to the inhibition of downstream *FT2a* and *FT5a*, and thus extremely late flowering. In the absence of phyA2 and phyA3, the LUX proteins are stable and can inhibit the transcription of *E1*. The small amount of *E1* that can be transcribed and translated also cannot exist stably because of the absence of phyA2 and phyA3, so the inhibition of *FT2a* and *FT5a* genes by E1 is released. *FT2a* and *FT5a* genes were transcribed, promoting flowering. When E1 and its homologs are missing, the transcription and post-translational regulations to E1 are failed to exist, result in release of E1 inhibition of *FT2a* and *FT5a*, and hence extremely early flowering. The red, orange, blue and purple circle represent phyA3, phyA2, E1 and LUX protein, respectively. Protein degradation was indicated as the circle replaced by smaller particles.

Dataset S1 (separate file). List of primer sequences used in this study.

SI References

1. S. Lu, *et al.*, Natural variation at the soybean J locus improves adaptation to the tropics and enhances yield. *Nat. Genet.* **49**, 773–779 (2017).
2. Z. Wu, *et al.*, RNA binding proteins RZ-1B and RZ-1C play critical roles in regulating Pre-mRNA splicing and gene expression during development in Arabidopsis. *Plant Cell* **28**, 55–73 (2016).
3. S. Shimizu-Sato, E. Huq, J. M. Tepperman, P. H. Quail, A light-switchable gene promoter system. *Nat. Biotechnol.* **20**, 1041–1044 (2002).
4. H. Zhang, *et al.*, SERK Family receptor-like kinases function as co-receptors with PXY for plant vascular development. *Mol. Plant* **9**, 1406–1414 (2016).
5. R. M. Galvão, *et al.*, Photoactivated phytochromes interact with HEMERA and promote its accumulation to establish photomorphogenesis in Arabidopsis. *Genes. Dev*. **26**, 1851–1863 (2012).
